# Machine learning model and nomogram to predict the risk of heart failure hospitalization in peritoneal dialysis patients

**DOI:** 10.1080/0886022X.2024.2324071

**Published:** 2024-03-17

**Authors:** Liping Xu, Fang Cao, Lian Wang, Weihua Liu, Meizhu Gao, Li Zhang, Fuyuan Hong, Miao Lin

**Affiliations:** a Department of Nephrology, The Second Affiliated Hospital of Xiamen Medical College, Xiamen, China; b Department of Nephrology, Provincial Clinical College, Fujian Medical University, Fujian Provincial Hospital, Fuzhou, Fujian, China; c Department of Nursing, Provincial Clinical College, Fujian Medical University, Fujian Provincial Hospital, Fuzhou, Fujian, China

**Keywords:** Peritoneal dialysis, machine learning, all-cause mortality, heart failure, complications

## Abstract

**Introduction:**

The study presented here aimed to establish a predictive model for heart failure (HF) and all-cause mortality in peritoneal dialysis (PD) patients with machine learning (ML) algorithm.

**Methods:**

We retrospectively included 1006 patients who initiated PD from 2010 to 2016. XGBoost, random forest (RF), and AdaBoost were used to train models for assessing risk for 1-year and 5-year HF hospitalization and mortality. The performance was validated using fivefold cross-validation. The optimal ML algorithm was used to construct the models to predictive the risk of the HF and all-cause mortality. The prediction performance of ML methods and Cox regression was compared.

**Results:**

Over a median follow-up of 49 months. Two hundred and ninety-eight patients developed HF required hospitalization; 199 patients died during the follow-up. The RF model (AUC = 0.853) was the best performing model for predicting HF, and the XGBoost model (AUC = 0.871) was the best model for predicting mortality. Baseline moderate or severe renal disease, systolic blood pressure (SBP), body mass index (BMI), age, Charlson Comorbidity Index (CCI) score were strongly associated with HF hospitalization, whereas age, CCI score, creatinine, age, high-density lipoprotein cholesterol (HDL-C), total cholesterol, baseline estimated glomerular filtration rate (eGFR) were the most significant predictors of mortality. For all the above endpoints, the ML models demonstrated better discrimination than Cox regression.

**Conclusions:**

We developed and validated a novel method to predict the risk factors of HF and all-cause mortality that integrates readily available clinical, laboratory, and electrocardiographic variables to predict the risk of HF among PD patients.

## Introduction

Peritoneal dialysis (PD) is one of the well-established renal replacement therapies that uses the peritoneal membrane for the liquid interchange; a catheter was placed in the peritoneal fundus through minor surgery or percutaneous technique. Peritoneal dialysis patients have many complications, among which cardiovascular disease is the most common cause of death in dialysis patients [1,2], and manifests as acute myocardial infarction, heart failure (HF), or sudden cardiac death, and EF preserved HF is one of the most frequent.

Cardiovascular disease is the main risk factor of death in maintenance dialysis patients, due to arteriosclerosis and chronic inflammation. Compared with hemodialysis patients, the use of glucose solution can lead to metabolic syndrome and obesity in PD patients. Because of this, the risk of dyslipidemia and hyperinsulinemia is higher. However, in terms of long-term survival, hemodialysis and PD are similar.

Despite current medical advances in the diagnosis and evidence-based management of HF, the outcome of HF remains unsatisfactory. This may be related to the poor accuracy of traditional medicine in predicting diseases. A number of clinical risk models based on linear models have been developed to predict early mortality in the dialysis patients, such as logistic or Cox model [3–7]. The Framingham Risk Score (FRS) is one of these commonly used clinical models [8]. In the research of HF, the Seattle Heart Failure Model is the most widely used risk prediction models, which achieved a *C*-statistic of 0.73 for mortality [9]. However, there are few studies in PD patients.

In recent years, machine learning (ML) has been proven to be a very powerful method in medical fields [10–13]. ML is a field of artificial intelligence, which mainly challenges big data and high dimensional data. The most prominent area of research which has seen rapid growth is in the field of diagnostics and prognosis [14–18]. ML, as a kind of classical prediction model, has good predictive performance [19]. By self-learning from big data, ML can produce a stable model that can predict the outcome of another dataset [20]. ML has been increasingly used also in the field of HF research [21,22]. HF is a significant health problem in PD patients worldwide. In the United States, the annual mortality rate of such patients is as high as 20.7%, and the median morbidity hospitalization is two times per year [23]. In this study, ML algorithm was used to build a model to predict the risk factors of HF and all-cause death in PD patients. Early diagnosis, early treatment, improve the quality of life.

## Materials and methods

### Study population

This study included patients who underwent PD catheterization and continuous ambulatory peritoneal dialysis (CAPD) in the Department of Nephrology, Fujian Provincial Hospital from January 2010 to December 2016. All adults aged ≧18 years. Patients were considered eligible for study inclusion when they had been on CAPD treatment for 3 months or more. Exclusion criteria included patients with underlying malignancy, pregnancy, severe mental illness, severe lung disease, severe heart disease (NYHA III–IV) or congenital heart disease, active stage of autoimmune disease requires large dose of glucocorticoid and immunosuppressive therapy, missing data >10%. In total, 1200 PD patients were enrolled in the study. Six patients with underlying malignancy, four patients with severe lung disease, 27 patients with severe heart disease (NYHA III–IV), and one patient with active stage of autoimmune disease requires large dose of glucocorticoid and immunosuppressive therapy and 18 patients had >10% missing data were excluded. One thousand one hundred and forty-four patients were included in this study. The participants were followed until February 2021. Forty patients transferred to hemodialysis, 23 patients received kidney transplantation, and 75 patients lost follow-up. The final analysis included 1006 participants. The participants were randomly assigned to development set and external validation cohort. The development set were 606 participants. The external validation cohort included 400 participants. The work flow of the patients’ inclusion procedure is presented in Suppl. Figure 1. This study was approved by the Fujian Provincial Hospital ethics committee as an exempt study with a waiver of informed consent, allowing a retrospective review of medical records.

### Data set

The data were obtained from the electronic medical records system and the laboratory information management system of the Fujian Provincial Hospital. All the data were collected for routine patient management with no additional data input required for the modeling. Clinical data of eligible patients were extracted and aggregated into a data tables for use by the ML models.

### The endpoints

The endpoints were defined as HF hospitalization and all-cause death. HF is a clinical syndrome characterized by a range of symptoms (dyspnea, limited physical activity, and edema) and signs (elevated jugular vein pressure, pulmonary congestion), usually caused by a structural or functional disease of the heart that results in ventricular filling and/or impaired ejection capacity. HF is classified according to New York Heart Association (NYHA) functional class. The primary end point was HF hospitalization, NYHA functional class III–IV. The secondary end point was all-cause death.

Diagnostic criteria for HF [24]: (1) dyspnea, fatigue, or decreased activity endurance; (2) signs of fluid retention (pulmonary congestion and lower limb swelling): such as elevated jugular venous pressure, wet rales at the bottom of lung; (3) abnormal cardiac structure and/or function in echocardiography and pulmonary edema indicated by imaging. (4) The symptoms, signs, and imaging manifestations of the above patients were relieved by intensive dialysis, which included increasing the dialysis dose or using high concentration peritoneal dialysate. Physicians choose intensive dialysis according to the patient’s peritoneal function and the degree of HF.

Dyspnea and fatigue were measured using four-point and five-point exertion scales recorded respectively by the investigator [25]. The investigator quantified the severity of lung congestion by Congestion Score Index (CSI) [26,27] and assessed the degree of peripheral edema at baseline [28].The above symptoms, signs, and imaging scoring systems were also used to evaluate the remission of HF. The details of scales of dyspnea, fatigue, and lung congestion, peripheral edema are shown in the Suppl. Data.

### Candidate variables

For each of the variable, selection was clinical routine data. In total, 113 patient characteristics (including demographics, comorbidities, vital signs, laboratory characteristics, electrocardiogram, echocardiogram, digital radiography, and therapy) were collected as variable candidates by medical record. Variables with >10% missing data were excluded, and removed variables with a correlation coefficient >0.5. Finally, the study analyzed 96 variables. Missing data <10% were imputed using a random forest (RF) imputation.

The Charlson Comorbidity Index (CCI) [29] includes 19 variables related to comorbidities, with scores ranging from 1 to 6, and the sum of ownership weights is the single complication score for each patient.

In accordance with the 2016 European Society of Cardiology Guidelines for HF, an echocardiogram has at least one of the following abnormalities: LA enlargement, LV hypertrophy, or mitral valve inflow/tissue Doppler abnormalities. Echocardiography was performed by experienced sonographers after 3 months PD. The two examiners of echocardiography have undergone uniform training.

Peritoneal equilibration test (PET): proposed by Twardowski in 1987. Corrected serum calcium (mmol/L) = Ca(mmol/L) + 0.02 × [40 – ALB (g/L)]. Estimated glomerular filtration rate (eGFR) was calculated by the CKD-EPI formula. Left ventricular mass (LVM) was calculated via Devereux formula [30]: LVM (g) = 0.80 [1.04 × (LVEDD + IVS + PWT)^3^ – LVED D^3^] + 0.6 g. The LVM index (LVMI) was calculated as LVM divided by the body surface area. Left ventricular hypertrophy (LVH) was defined as LVMI > 134 g/m^2^ in male and >110 g/m^2^ in female [31,32]. Ejection fraction (EF) was grouped by 50% (where ALB: albumin; Ca: calcium; LVEDD: left ventricular end diastolic dimension; IVS: interventricular septum; PWT: posterior wall thickness; LV: left ventricular; LA: left atrial).

### Statistical analysis

Baseline data were analysis by SPSS 22.0 software (SPSS Inc., Chicago, IL). Baseline data were displayed as mean ± standard deviation or median (interquartile range) for continuous variables and as percentages for categorical variables. The normal distribution variables and non-normal distribution variables were tested using the *t*-test or Mann–Whitney’s *U*-test. The counting data were expressed by percentage and *χ*^2^ test. *p* < .05 was considered statistically significant. Homogeneity of variance analysis and Pearson’s correlation test were performed on statistically significant variables. Variables of VIF > 10 and correlations > 0.5 were eliminated.

### Model development (RF-based and XGB-based selection)

Three different ML algorithms were considered: XGBoost, RF, and AdaBoost. The performance of the models was assessed using fivefold cross-validation. Eighty percent of the data were used as training set and 20% as validation set. All data were randomly divided into five subsets of similar size and mutually exclusive. Each round of training selects four subsets to form the training set and the rest subsets to form the validation set. Each model required training and validation five times, with different training and validation sets being used each time. The average of the five test results was accepted as the final result. The ML algorithm with the highest AUC value was selected for modeling. The important variables affecting the outcome were output and rank. The optimal model is output using the following measures: area under the ROC curve (AUC), accuracy, sensitivity, specificity, positive predictive value (PPV), negative predictive value (NPV), and *F*1-score. Compared with the prediction performance of optimal ML algorithm and Cox regression, all statistical analysis was done using R language 3.6.3 and python 3.7 environment (R Foundation for Statistical Computing, Vienna, Austria).

## Results

### Baseline characteristics

Among the development set, the mean (±SD) age was 52.6 ± 16.1 years, 63.67% of patients were male. The median follow-up time was 49 months (IQR 16–69). 60.07% of patients had chronic glomerulonephritis, 25.41% of patients had diabetes, and 1.82% of patients had hypertension. The remaining kidney diseases included polycystic kidney disease and obstructive kidney disease, accounting for 12.71% (shown in Table 1, Suppl. Tables 1 and 2).

**Table 1.** Baseline data table for heart failure endpoints.

Characteristics	All patients (*n* = 606)	No HF event during follow-up (*n* = 308)	Incident HF event during follow-up (*n* = 298)	*p* Value
Age (years), mean (SD)	52.6 ± 16.1	50.7 ± 15.9	54.5 ± 16.1	.005
Female, *n* (%)	222 (36.6)	121 (39.3)	101 (33.9)	.168
Dialysis duration (months), mean (SD)	47.9 ± 33.6	62.3 ± 34.3	33.1 ± 25.7	<.001
Smoking history, *n* (%)	130 (21.5)	56 (18.2)	74 (24.8)	.046
BMI (kg/m^2^), mean (SD)	22.6 ± 3.1	22.2 ± 2.9	23.0 ± 3.2	.003
SBP (mmHg), mean (SD)	149.2 ± 21.6	147.4 ± 21.3	151.1 ± 21.8	.016
DBP (mmHg), mean (SD)	83.6 ± 14.4	84.6 ± 14.3	82.6 ± 14.3	.100
Daily urine volume (ml), mean (SD)	807.5 ± 512.5	855.7 ± 515.6	757.7 ± 505.5	.009
Total weekly *Kt*/*V*, mean (SD)	1.8 ± 0.5	1.8 ± 0.5	1.9 ± 0.5	.628
eGFR (ml/min/1.73 m^2^), mean (SD)	6.0 ± 4.2	6.0 ± 4.8	5.9 ± 3.6	.611
PET, mean (SD)	0.74 ± 0.15	0.74 ± 0.16	0.73 ± 0.15	.649
Primary renal disease, *n* (%)				
Glomerulonephritis	364 (60.1)	208 (67.5)	156 (52.3)	<.001
Diabetes	154 (25.4)	50 (16.2)	104 (34.9)	<.001
Hypertension	11 (1.8)	4 (1.3)	7 (2.3)	.333
Others	77 (12.7)	46 (14.9)	31 (10.4)	.094
Hypertension, *n* (%)	554 (91.4)	275 (89.3)	279 (93.6)	.0057
CCI score, mean (SD)	5.1 ± 2.7	4.4 ± 2.3	5.9 ± 2.9	<.001
Biochemical parameters				
Hemoglobin (g/l), mean (SD)	86.4 ± 19.9	85.0 ± 19.9	87.9 ± 19.9	.073
Serum albumin (g/l), mean (SD)	30.4 ± 7.0	30.3 ± 7.1	30.4 ± 7.0	.666
Serum calcium (mmol/l), mean (SD)	2.9 ± 0.5	2.9 ± 0.5	2.9 ± 0.48	.440
Serum phosphorus (mmol/l), mean (SD)	1.9 ± 0.7	1.9 ± 0.7	1.9 ± 0.6	.318
Serum intact PTH (pg/ml), median (IQR)	204.5 [106.2, 349.6]	206.6 [106.9, 363.3]	204.4 [106.2, 331.2]	.304
NT-proBNP (pg/ml), median (IQR)	9948.0 [2401.0, 33713.0]	10047.0 [2762.0, 31811.0]	9628.0 [2320.0, 34716.0]	.929
Echocardiographic parameters, mean (SD)				
EF (%)	59.4 ± 7.5	59.5 ± 7.1	59.4 ± 8.0	.248
Left ventricular diastolic volume index (ml/m^2^)	62.7 ± 19.9	61.7 ± 20.8	63.6 ± 19.2	.887
Right ventricular diameter (cm)	6.4 ± 0.6	3.4 ± 0.6	3.4 ± 0.6	.707
LVPWT (cm)	1.1 ± 0.3	1.1 ± 0.4	1.1 ± 0.2	.272
IVST (cm)	1.2 ± 0.2	1.2 ± 0.2	1.3 ± 0.2	.187
LVMI (g/m^2^), mean (SD)	142.0 ± 48.4	140.4 ± 44.2	143.5 ± 52.0	.465
Electrocardiogram ST-T change, *n* (%)	293 (48.4)	129 (41.9)	164 (55.0)	.001
Cardiac enlargement on DR, *n* (%)	127 (21.0)	51 (16.6)	76 (25.5)	.007

SD: standard deviation; NT-pro-BNP: N-terminal pro-brain natriuretic peptide; BMI: body mass index; SBP: systolic blood pressure; DBP: diastolic blood pressure; PD: peritoneal dialysis; eGFR: estimated glomerular filtration rate; PET: peritoneal equilibration test; ESRD: end-stage renal disease; CCI: Charlson Comorbidity Index; PTH: parathyroid hormone; EF: ejection fraction; LVPWT: left ventricular diastolic posterior wall thickness; LVMI: left ventricular mass index; IVST: interventricular septum thickness.

Data are presented as percentages, median (interquartile range) or mean ± SD. *p* Values <.05 were considered statistically significant.

### Results for predicting the risks of HF hospitalization

**Figure 1.**
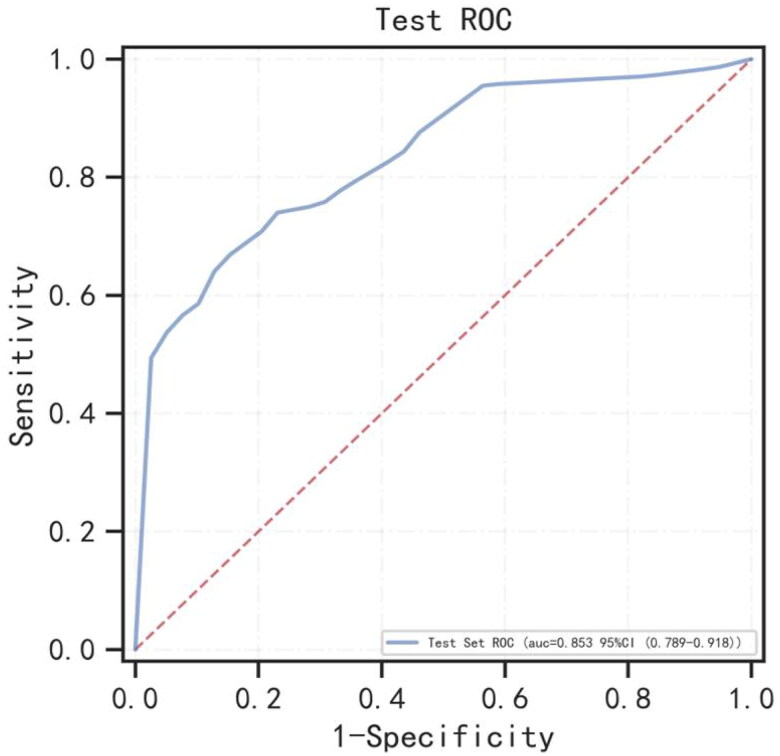
The ROC curve for the random forest model for predicting heart failure in the test set. AUC: area under the curve.

**Figure 2.**
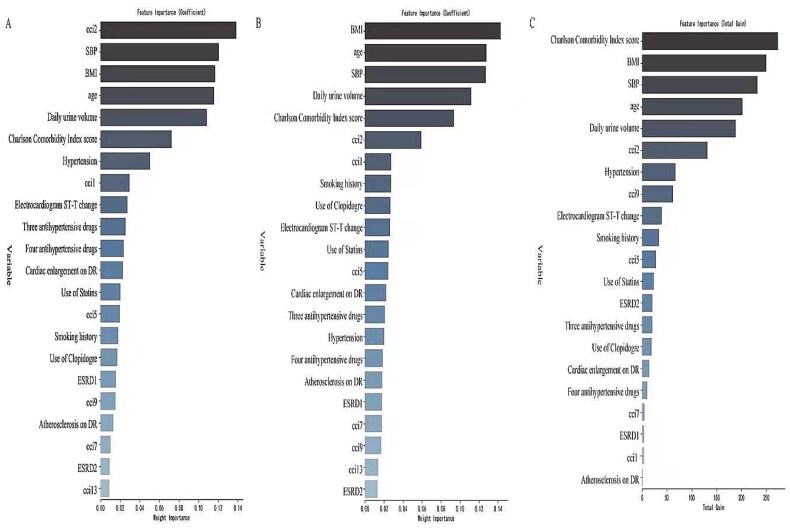
Variable importance analysis. Results indicate the decrease in accuracy of the final model on exclusion of each specific variable, quantified on a scale of 0 to 0.14, 250, respectively. While 0 represents the minimum importance (lowest decrease in the accuracy when excluded), 0.14 and 250 represent the maximum importance (highest decrease in the accuracy when excluded). (A) Variable importance analysis of heart failure. (B) Variable importance analysis of 1-year heart failure. (C) Variable importance analysis of 5-year heart failure; CCI1: myocardial infarction; CCI2: congestive heart failure; CCI5: dementia; CCI7: connective tissue disease/rheumatic disease; CCI9: mild liver disease; CCI13: renal disease. ESRD1: primary glomerulonephritis; ESRD2: diabetes; SBP: systolic blood pressure; BMI: body mass index.

In this study, 298 of 606 patients developed HF required hospitalization during follow-up. As many as 65% of patients with HF were left ventricular EF preserved. Twenty-four significant variables were choosen from baseline characteristics (shown in Table 1 and Suppl. Table 1). The performance of XGBoost, RF, and AdaBoost were assessed using fivefold cross-validation, the AUC values of training set were 0.844, 0.931, and 0.821, respectively (shown in Suppl. Figure 2(A)), the AUC values of validation set were 0.793, 0.794, and 0.794, respectively (shown in Suppl. Figure 2(B)). Finally, the optimal model of RF with the highest AUC was selected for modeling. Each index of the optimal model in the test set and the test set ROC curve are presented in Table 2 and Figure 1. Figure 2(A) presents the risk variables for predicting HF from the RF model. CCI2 (the history of congestive HF) was the most important risk factor. Systolic blood pressure (SBP), body mass index (BMI), and other risk factors followed.

**Table 2.** Performance each index of the optimal model in the test set of AUC, accuracy, sensitivity, specificity, PPV, NPV, and *F*1-score.

End point	Machine learning algorithm	AUC	Accuracy	Sensitivity	Specificity	PPV	NPV	*F*1-score
HF	Random forest	0.853	0.746	0.710	0.849	0.788	0.696	0.747
1-Year follow-up HF	Random forest	0.729	0.739	0.923	0.575	0.179	0.912	0.299
5-Year follow-up HF	XGBoost	0.698	0.639	0.655	0.738	0.692	0.578	0.673
All cause death	Random forest	0.871	0.821	0.780	0.892	0.806	0.829	0.793
1-Year all cause death	XGBoost	0.669	0.765	0.857	0.582	0.056	0.925	0.104
5-Year all cause death	Random forest	0.829	0.743	0.765	0.806	0.722	0.765	0.743

AUC: area under the curve; PPV: positive predictive value; NPV: negative predictive value.

### Results for predicting the risks of the first year follow-up HF hospitalization

**Figure 3.**
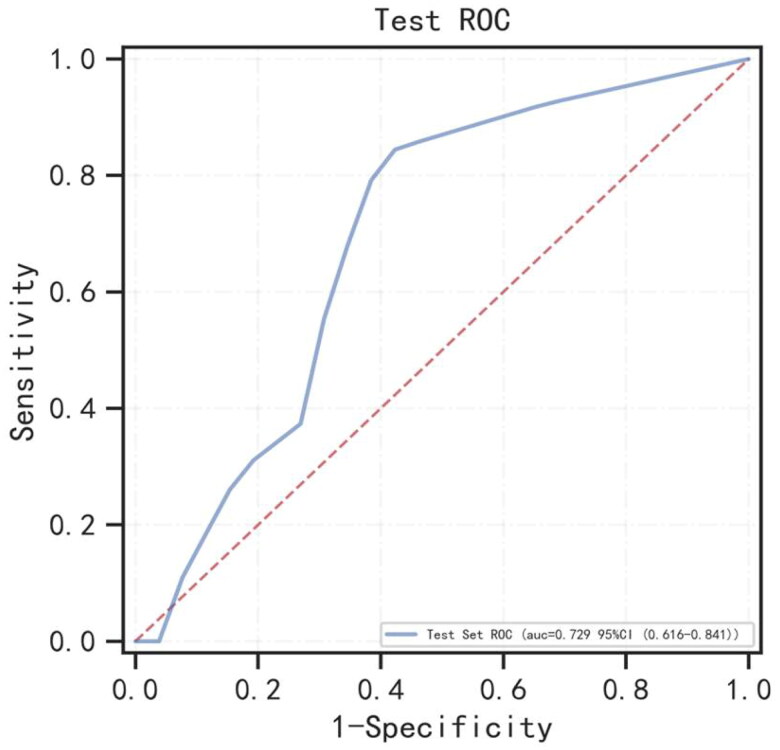
The ROC curve for the random forest model for predicting heart failure at year 1 in the test set. AUC: area under the curve.

In this sub-endpoint, during the follow-up, 35 patients died within 1 year without HF. Seventy-nine of the 571 patients developed HF required hospitalization during the first year follow-up. Twenty-four significant variables were chosen from baseline characteristics (shown in Table 1 and Suppl. Table 1). The performance of XGBoost, RF and AdaBoost was assessed using fivefold cross-validation, the AUC values of training set were 0.874, 0.943, and 0.840, respectively (shown in Suppl. Figure 3(A)), the AUC values of validation set were 0.691, 0.728, and 0.704, respectively (shown in Suppl. Figure 3(B)). Finally, the optimal model of RF with the highest AUC was selected for modeling. Each index of the optimal model in the test set and the test ROC curve are presented in Table 2 and Figure 3. Figure 2(B) presents the risk variables for predicting the HF in first year follow-up from the RF model. BMI was the most important risk factor. Age, SBP, and other risk factors followed.

### Results for predicting the risks of the 5-year follow-up HF hospitalization

**Figure 4.**
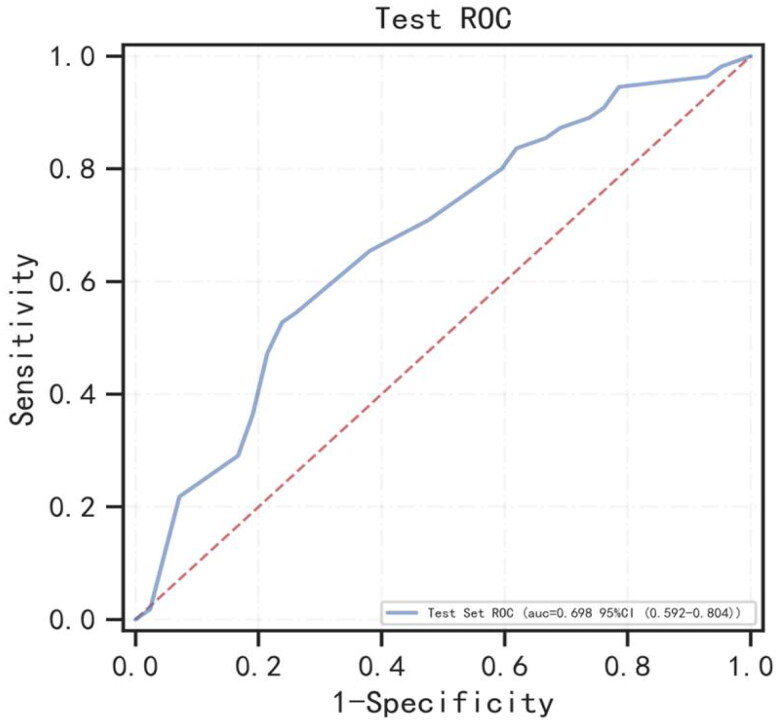
The ROC curve for the XGBoost model for predicting heart failure at year 5 in the test set. AUC: area under the curve.

In this sub-endpoint, during the follow-up, 150 patients died within 5 year without HF. Two hundred and forty-six of the 456 patients developed HF during 5-year follow-up. Twenty-four significant variables were chosen from baseline characteristics (shown in Table 1 and Suppl. Table 1). The performance of XGBoost, RF, and AdaBoost was assessed using fivefold cross-validation, the AUC values of training set were 0.944, 0.806, and 0.857, respectively (shown in Suppl. Figure 4(A)), the AUC values of validation set were 0.706, 0.703, and 0.685, respectively (shown in Suppl. Figure 4(B)). Finally, the optimal model of XGBoost with the highest AUC was selected for modeling. Each index of the optimal model in the test set and the test ROC curve are presented in Table 2 and Figure 4. Figure 2(C) presents the risk variables for predicting the HF in 5-year follow-up from the XGBoost model. CCI score was the most important risk factor. BMI, SBP, and other risk factors followed.

### Comparison of predictive performance between Cox and ML model in HF hospitalization endpoint

In this study, Cox model was used to evaluate the influence of risk variables in the endpoint of HF. The *C*-index of the Cox model in training set was 0.668 and test set was 0.724. The AUC of ML optimal model in test set was 0.853. In the 1-year and 5-year follow-up HF endpoints, the AUC of ML optimal model in test set was 0.729 and 0.698, respectively, while the AUC of Cox were 0.723 and 0.759 (shown in Suppl. Figure 5(A,B)).

### Nomogram for predicting HF

**Figure 5.**
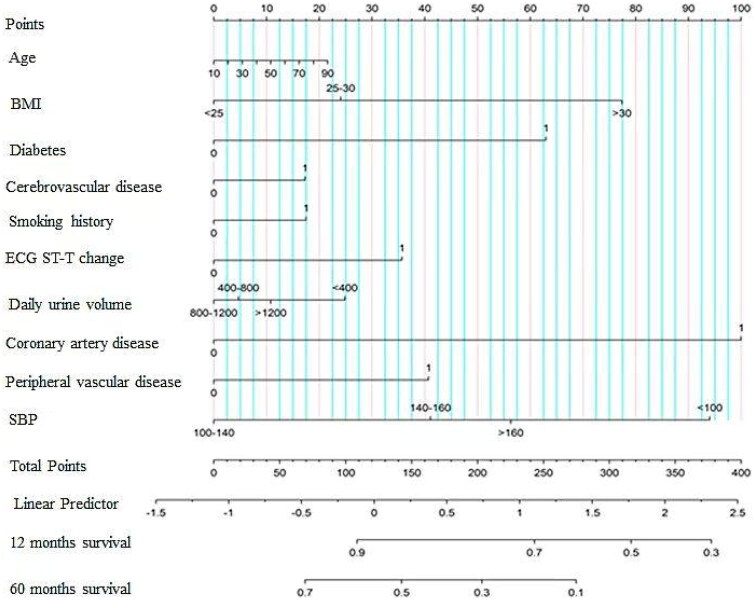
Nomograms based on heart failure endpoints. The nomogram was quantified according to the weights of the variables selected by the model. BMI: body mass index; ECG: electrocardiograph; SBP: systolic blood pressure.

The ML model analysis contained risk variables for HF in PD patients, including categorical variables generated from continuous variables. The nomogram was quantified according to the weights of the variables selected by the model. In the nomogram analysis, age > 90, BMI > 30, SBP <100 mmHg, daily urine volume < 100, coronary artery disease, peripheral vascular disease, diabetes, ECG ST-T change, congestive HF, and smoking history were independent risk factors associated with HF hospitalization. Based on the ML model, a nomogram was plotted to predict the probability of HF hospitalization in PD patients (Figure 5).

### Risk score system for HF incidence

From the 10 identified top-performing HF predictors, a risk score system for HF incidence was created (shown in Suppl. Figure 6), the risk variables were age, BMI, SBP, smoking history, congestive HF, daily urine volume, coronary artery disease, peripheral vascular disease, diabetes, and electrocardiogram ST-T change. The risk score ranges from 0 to 56. The observed scores range from 0 to > 38. The risk of HF increased with the increase of the score at 1 and 5 years of follow-up. A one-classification increment in the risk score was associated with a 20% higher risk of HF at 1-year or 5-year. It is divided into three risk levels according to risk scores (risk = 0, risk = 1, and risk = 2).

### External validation of the risk score system for HF incidence

**Figure 6.**
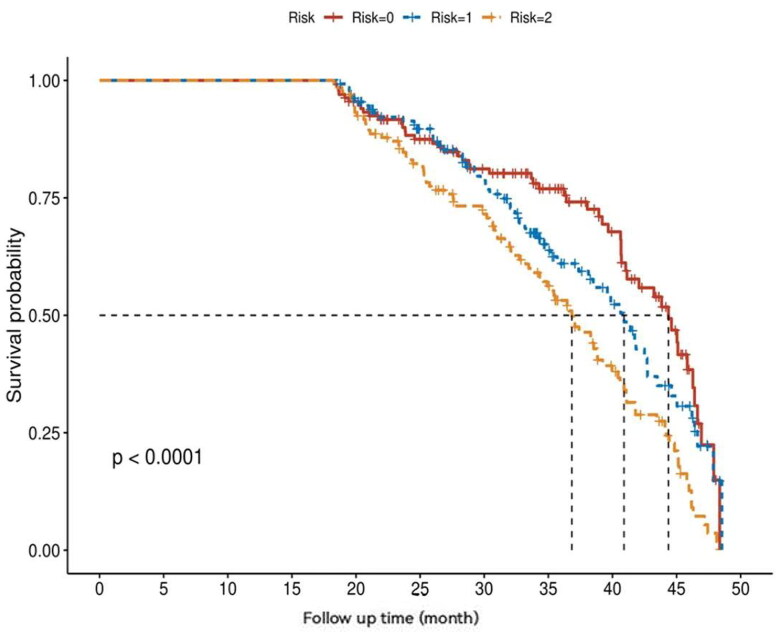
External validation of the risk score system for HF incidence. Kaplan–Meier’s survival curves for PD patients with higher and lower risks of HF. The risk score is divided into three levels. The survival prognosis of PD patients in the high-risk group was significantly worse than that in the low-risk group follow-up time (m): the time from study enrollment to the end of HF.

The risk score system for HF incidence was externally validated in the subgroup of PD patients at baseline. The external validation cohort included 400 PD patients with 208 incident HF events (52%). After plotting the externally verified survival curve, we found that the survival prognosis of PD patients in the high-risk group was significantly worse than that in the low-risk group (shown in Figure 6). The risk score system also had good risk prediction performance.

### Results for predicting the risks of all cause death

Regarding all-cause mortality, 199 of 606 patients died during the follow-up. Forty-four significant variables were choosen from baseline characteristics (shown in Suppl. Table 2). The performance of XGBoost, RF, and AdaBoost was assessed using fivefold cross-validation, the AUC values of training set were 0.897, 0.935, and 0.816, respectively (shown in Suppl. Figure 7(A)), the AUC values of validation set were 0.828, 0.829, and 0.746, respectively (shown in Suppl. Figure 7(B)). Finally, the optimal model of RF with the highest AUC was selected for modeling. Each index of the optimal model in the test set and the test ROC curve are presented in Table 2 and Suppl. Figures 7(C) and 8(A) presents the risk variables for predicting the all-cause death endpoint from the RF model. Age was the most important risk factor. CCI score, CR, and other risk factors followed.

### Results for predicting the risks of the 1-year all cause death

In this study, 64 of 606 patients died during the first year follow-up. Forty-four significant variables were chosen from baseline characteristics (shown in Suppl. Table 2). The performance of XGBoost, RF, and AdaBoost was assessed using fivefold cross-validation, the AUC values of training set were 0.876, 0.807, and 0.753, respectively (shown in Suppl. Figure 9(A)), the AUC values of validation set were 0.709, 0.667, and 0.667, respectively (shown in Suppl. Figure 9(B)). Finally, the optimal model of XGBoost with the highest AUC was selected for modeling. Each index of the optimal model in the test set and the test ROC curve are presented in Table 2 and Suppl. Figure 9(C) and 8(B) presents the risk variables for predicting the 1-year all cause death from the XGBoost model. Age was the most important risk factor. High-density lipoprotein cholesterol (HDL-C), total cholesterol, and other risk factors followed.

### Results for predicting the risks of the 5-year all cause death

In this study, 161 of 606 patients died during 5-year follow-up. Forty-four significant variables were chosen from baseline characteristics (shown in Suppl. Table 2). The performance of XGBoost, RF, and AdaBoost was assessed using fivefold cross-validation, the AUC values of training set were 0.899, 0.938, and 0.832, respectively (shown in Suppl. Figure 10(A)), the AUC values of validation set were 0.795, 0.809, and 0.774, respectively (shown in Suppl. Figure 10(B)). Finally, the optimal model of RF with the highest AUC was selected for modeling. Each index of the optimal model in the test set and the test ROC curve are presented in Table 2 and Suppl. Figure 10(C) and 8(C) presents the risk variables for predicting the 5-year all cause death from the RF model. Age was the most important risk factor. CCI score, eGFR, and other risk factors followed.

### Comparison of predictive performance between Cox and ML model in all cause death endpoint

In this study, Cox model was used to evaluate the influence of risk variables in the endpoint of all cause death. The *C*-index of the Cox model in training set was 0.758 and test set was 0.769. The AUC of ML optimal model in test set was 0.871. In the 1-year and 5-year follow-up all cause death endpoints, the AUC of ML optimal model in test set were 0.669 and 0.829, respectively, while the AUC of Cox were 0.789 and 0.804 (shown in Suppl. Figure 11(A,B)).

## Discussion

The purpose of our study was accurately predicting the risks of HF hospitalization and all cause death in PD patients using ML tools. To our knowledge, this is the latest study to predict the risks of HF hospitalization and all cause death in PD patients using various ML algorithms. Our ML predicting models have several unique characteristics. First, ML has shown its advantages in the processing of big medical data, especially suitable for the study of PD patients with complex complications. Second, data noise and missing data are inevitable in data collecting from the real world, especially the retrospective studies, and ML algorithm can easy handle the complex problem. Third, compared to the traditional models, ML models allow data to be constantly updated and can accurately capture feature connections between data.

In this study, age was an independent risk variable for all-cause death and HF in PD patients. Aging is a major risk factor for cardiovascular disease and a prognostic determinant of chronic HF [33,34]. According to the risk factors which derived from the ML model, we suggest reducing the age-related comorbidities index, which may help select patients in PD. The Framingham study showed that for every additional 10 years of age, the rate of HF increases twofold. PD patients have many complications, even absence of other comorbidities, weakness associated with aging increased mortality. We also propose thresholds for age-related comorbidities index, which may help evaluate the risk of HF and death in PD patients.

Another outstanding advantage of our study is that ML algorithm has increased usefulness of the CCI. Among the chronic disease indices validated in the dialysis patients, CCI has been widely used in statistical analyses due to its simplicity and ability to predict mortality, the original CCI has been validated in PD patients [35,36]. PD patients have complicated comorbidities, so CCI is quantifiable to evaluate the comorbidities. In this study, CCI score had a high weight in predicting the risk of all cause death and HF in PD patients, which was consistent with relevant studies.

Pulliam et al. [37] conducted a study on PD patients in North America and found that the mortality of PD patients was 10% within one year. In our study, during the first year follow-up, the mortality was 11.5%, the result is similar with relation studies. According to a 5-year follow-up report released by the American Kidney Disease Data System in 2015 since 2008 [38], the 3-year survival rate of PD patients was 66.4%, and the 5-year survival rate was 50.3%, with the mortality decreasing year by year. In our ML algorithm, the risks of 5-year all-cause mortality were age, CCI score, eGFR, creatinine, right ventricular diameter, DBP, total cholesterol, HDL-C, ALB, etc. It shows that, as longer as the duration of dialysis, complications, dialysis adequacy, blood lipid, and nutritional affect the survival.

One of the most widely used risk prediction models of HF is the Seattle HF model, which obtained an AUC value of 0.73 [9]. However, few studies have used ML methods to predict the risk factors of HF in PD patients; this is another highlight of our study. Recent data show that ML algorithms outperform logistic regression models in the prediction of HF outcomes [39–41].The traditional indicators of assessing HF such as edema, pulmonary rales, BNP were not sensitive and lagging. ML model has high sensitivity and specificity for clinical risk predicting. In our study, the AUC value of the risk predict model is above 0.8. In comparison with the Cox model, ML is mostly better than the Cox model. However, in the partial time endpoints, the ML model does not show superiority, which is related to the sample size. In this study, NT-proBNP and EF in predicted HF showed no significant difference, it may be because in patients with renal failure and insufficiency, NT-proBNP metabolism is slow. As for EF in echocardiography, although the average EF of the HF group was slightly lower than that of the non-HF group, due to insufficient sample size, the difference was not statistically significant.

This ML-based approach can deal with large multidimensional time-to-data sets, it does not need to consider whether the data are normally distributed or not, and the over-fitting of the model. Based on the ML algorithm, we have also developed an integer-based risk score, which can effectively predict the risk of HF in PD patients. From the risk scoring system, the PD patients with higher scores show an upward trend with the increase of scores during 1-year and 5-year follow-up. We can develop a risk scoring software for clinical use to evaluate the risk and survival of HF in PD patients.

To conclude, the purpose of our study was using ML algorithmic to accurately predict the risks of HF and all-cause mortality in PD patients by focusing on comorbidities and clinical information. Our models could help informing patients and caregivers early detection of the HF risk and all-cause mortality risk and may aid decision making in PD patients who are faced with the complication. Our risk scoring system is also applicable to the clinical evaluation of the HF risk of PD patients.

## Study strengths and limitations

Our study has several strengths. The advantage of this study is that although ML has been applied in the clinical use and even in the field of dialysis, the research on PD is still lacking. This is the first ML study of all-cause mortality and cardiovascular complications in PD patients.

However, the limitations of our study are also worth mentioning. First, our study is a retrospective, single-center study with a limited sample size. Second, ML algorithms fail to explain the relationship between variables and endpoints. Third, the gold standard for PD capacity evaluation is isotope dilution, and dual-energy X-ray and bioelectrical impedance, but these methods cannot be used as a routine evaluation method for large-scale clinical studies due to operational limitations. It is impossible to distinguish between water and sodium retention or cardiac dysfunction by the traditional indicators in our study, especially in cases of normal EF. Fourth, this study only recorded HF patients requiring hospitalization, but did not include patients with subclinical symptoms of HF. Fifth, the scoring system needs to be verified by a larger sample size. In addition, some variables were excluded due to the high degree of deletion in our cohort, especially the echocardiography data, which can be continued in the future study.

## Conclusions

We implemented ML algorithms to accurately predict the risks of HF and all-cause mortality in PD patients. Finally, we provide comprehensive results of our study using different ML algorithms. Our study suggested potential of ML to facilitate risk stratification and to discover new predictors in complex clinical situations, such as PD patients with many complications. This ML approach proved to have comparable forecasting value with Cox regression and was better than Cox model.

## Supplementary Material

Supplemental Material

## Data Availability

Data are not publicly available due to ethical reasons. Further enquiries can be directed to the corresponding author.
