# Supplementary material for: Machine learning model and nomogram to predict the risk of heart failure hospitalization in peritoneal dialysis patients
Source: Ren Fail. 2024 Mar 17;46(1):2324071. doi: 10.1080/0886022X.2024.2324071 (PMC10946267; doi:10.1080/0886022X.2024.2324071)
Supplement: Supplemental Material [file IRNF_A_2324071_SM4399.pdf]

Machine learning model and nomogram to predict the risk of heart failure hospitalization in peritoneal dialysis patients

Scales of dyspnea and fatigue

Dyspnea was measured using a 4-point exertion scale recorded by the investigator, as follows:1 point=heavy exertion, 2 points=moderate exertion, 3 points=slight exertion, 4 points=rest； fatigue was measured using a 5-point exertion scale recorded by the investigator, as follows:0=none, 1 point=heavy exertion, 2 points=moderate exertion, 3 points=slight exertion, 4 points=rest. Greater than 3 points scale in dyspnea or fatigue at baseline were exclude because of the exclusion criteria.

Scales of lung congestion and peripheral edema

The investigator quantified the severity of lung congestion by congestion score index (CSI) in baseline data. Lung field was divided into six areas, each area was evaluated as follows: Score 0, normal; Score 1, cephalization (superior area), perivascular/peribronchial cuffing or perihilar haze or Kerley’s A lines (middle area), Kerley’s B lines or Kerley’s C lines (inferior area); Score 2, interstitial or mild pulmonary edema; Score 3, intense pulmonary edema.

The investigator assessed the degree of peripheral edema at baseline and included (none, mild [1+] or marked [≧2+]; dichotomized as present [≧1+] or absent).

Suppl table1. Baseline data table for heart failure endpoints

characteristics	All patients(n=606)	No HF event during follow-up(n=308)	Incident HF event during follow-up(n=298)	P-value
Marriage,n(%)				
Discoverture	30(5.0)	20(6.5)	10(3.4)	0.075
Married	576(95.1)	288(93.5)	288(96.6)	0.075
General Educational Development,n(%)				
Primary and below	244(40.3)	133(43.2)	111(37.2)	0.136

Suppl table1 （continued）

characteristics	All patients(n=606)	No HF event during follow-up(n=308)	Incident HF event during follow-up(n=298)	P-value
Primary school or above	362(59.7)	175(56.8)	187(62.8)	0.136
Health care type,n(%)				
Self-paying	90(14.9)	54(17.5)	36(12.1)	0.059
Medicare reimbursement	517(85.3)	255(82.8)	262(87.9)	0.075
Employed field ,n(%)				
Unemployed	529(87.3)	281(91.2)	248(83.2)	0.003
Employed	77(12.7)	27(8.8)	50(16.8)	0.003
Used dialysate volume,（L）, mean (SD)	6.0±2.1	6.0[4.0,8.0]	6.0[4.0,8.0]	0.473
Daily net PD ultrafiltration volume,（ml）, mean (SD)	651.9±433.5	614.97±391.54	690.1±470.6	0.102
Charlson Comorbidities,(%)				
cci1 Coronary artery disease	59(9.7)	2(0.6)	57(19.1)	<0.001
cci2 Congestive heart failure	136(22.4)	8(2.6)	128(43.0)	<0.001
cci3 Chronic lung disease	41(6.8)	21(6.8)	20(6.7)	0.958
cci4 Peptic ulcer disease	27(4.5)	15(4.9)	12(4.0)	0.615
cci5 Peripheral vascular disease	173(28.5)	64(20.8)	109(36.6)	<0.001
cci6 Hepatic disease (unspecified)	83(13.7)	45(14.6)	38(12.8)	0.506
cci7 Cerebrovascular disease	84(13.9)	33(10.7)	51(17.1)	0.023
cci8 Connective tissue disorder	15(2.5)	9(2.9)	6(2.0)	0.472
cci9 Diabetes	159(26.2)	52(16.9)	107(35.9)	<0.001
cci10 Dementia	3(0.5)	2(0.7)	1(0.3)	
cci11 Hemiplegia or paraplegia	20(3.3)	7(2.3)	13(4.4)	0.150
cci12 Moderate or severe renal disease	606(100.0)	308(100.0)	298(100.0)	

Suppl table1 （continued）

characteristics	All patients(n=606)	No HF event during follow-up(n=308)	Incident HF event during follow-up(n=298)	P-value
cci13 Diabetes with chronic complication	127(21.0)	48(15.6)	79(26.5)	<0.001
cci14 Any malignancy in five years	36(5.9)	19(6.2)	17(5.7)	0.809
cci15 Leucocythemia	0(0)	0(0)	0(0)	1.000
cci16 Lymphoma	1(0.2)	1(0.3)	0(0.0)	
cci17 Moderate or severe liver disease	19(3.1)	7(2.3)	12(4.0)	0.215
cci18 Metastatic solid tumor	1(0.2)	1(0.3)	0(0.0)	
cci19 AIDS	0(0)	0(0)	0(0)	
ESA users,n(%)	535(88.3)	275(89.3)	260(87.2)	0.436
Use of VDRA,n(%)	262(43.2)	137(44.5)	125(41.9)	0.529
Use of Aspirin,n(%)	24(4.0)	8(2.6)	16(5.4)	0.080
Use of Clopidogrel,n(%)	160(26.4)	54(17.5)	106(35.6)	<0.001
Use of Statins,n(%)	204(33.7)	86(27.9)	118(39.6)	0.002
Use of Beta blockers,n(%)	340(56.1)	165(53.6)	175(58.7)	0.201
Use of CCB,n(%)	526(86.8)	261(84.7)	265(88.9)	0.128
Use of ACEI,n(%)	100(16.5)	46(14.9)	54(18.1)	0.291
Use of ARB,n(%)	174(28.7)	81(26.3)	93(31.2)	0.182
No. of antihypertensive drugs,n(%)				
One	143(23.6)	82(26.6)	61(20.5)	0.074
Two	159(26.2)	86(27.9)	73(24.5)	0.338
Three	156(25.7)	68(22.1)	88(29.5)	0.036
Four	78(12.9)	28(9.1)	50(16.8)	0.005

Suppl table1 （continued）

characteristics	All patients(n=606)	No HF event during follow-up(n=308)	Incident HF event during follow-up(n=298)	P-value
Five	18(3.0)	11(3.6)	7(2.3)	0.376
Phosphate binders use,n(%)				
Did not take medicine	314(51.8)	162(52.6)	152(51.0)	0.695
Calcium phosphorus binding agent	276(45.5)	139(45.1)	137(46.0)	0.835
Non calcium phosphorus binder	16(2.6)	7(2.3)	9(3.0)	0.566
Biochemical parameters				
Blood urea nitrogen（mmol/l）, mean (SD)	23.9±11.2	24.4±11.3	23.5±11.0	0.225
Serum creatinine（umol/l）, mean (SD)	899.6±353.1	903.9±343.4	895.2363.4	0.529
Uric acid（umol/l）, mean (SD)	461.0±139.0	455.8±138.2	468.1±141.3	0.370
LDL-C（mmol/l）, mean (SD)	2.7±1.1	2.7±1.0	2.8±1.2	0.353
HDL-C（mmol/l）, mean (SD)	1.1±0.4	1.1±0.4	1.1±0.4	0.809
Total cholesterol（mmol/l）, mean (SD)	4.6±1.5	4.5±1.3	4.6±1.7	0.542
Triglycerides（mmol/l）, mean (SD)	1.8±1.4	1.7±1.1	1.9±1.6	0.060
Cystatin-C（mg/l）, mean (SD)	6.0±1.9	6.0±1.9	5.9±1.9	0.702
Serum ferritin（ng/ml）, mean (SD)	390.9±331.9	427.7±359.5	438.0±385.9	0.978
Cardiac troponin T（ng/ml）,median (IQR)	0.043[0.02, 0.1]	0.046[0.02, 0.10]	0.04[0.02, 0.10]	0.339
PCT（ng/ml）,median (IQR)	0.5[0.2, 1.9]	0.5[0.2, 2.0]	0.5[0.2, 1.7]	0.947

Suppl table1 （continued）

characteristics	All patients(n=606)	No HF event during follow-up(n=308)	Incident HF event during follow-up(n=298)	P-value
CRP（mg/l）,median (IQR)	14.7[4.1,48.1]	14.1[4.4,48.0]	16.4[3.7,48.1]	0.854
Echocardiographic parameters, mean (SD)				
Right Atrium diameter（cm）	3.6±1.0	3.6±1.3	3.6±0.5	0.728
Pulmonary artery diameter（cm）	2.4±0.4	2.4±0.4	2.5±0.4	0.513
Left ventricular diameter（cm）	5.0±0.7	4.9±0.7	5.1±0.7	0.757
Left atrial dimension（cm）	3.9±0.6	3.8±0.6	3.9±0.6	0.598
Sinus of aorta（cm）	3.4±0.4	3.4±0.5	3.4±0.4	0.484
LVH on ECG, n(%)	108(17.8)	51(16.6)	57(19.1)	0.409
Auricular fibrillation on ECG,n(%)	11(1.8)	7(2.3)	4(1.3)	0.391
Atherosclerosis on DR,n(%)	111(18.3)	47(15.3)	64(21.5)	0.048

Data are presented as percentages, median (interquartile range) or mean±SD. SD, standard deviation; cci, Charlson-comorbidity index; AIDS, acquired immunodeficiency syndrome; ESA,erythropoiesis-stimulating agents; VDRA,vitamin D receptor activator; CCB, calcium channel blocker; ACEI, angiotension converting enzyme inhibitors; ARB, Angiotensin Receptor Blocker; LDL-C, low-density lipoprotein cholesterol; HDL-C, high- density lipoprotein cholesterol; CRP, C-reactive protein; PCT, Procalcitonin; ECG, electrocardiograph; LVH, left ventricular hypertrophy. P values <0.05 were considered statistically significant.

Suppl table2. Baseline data table for all-cause death endpoints

characteristics	All patients(n=606)	Non death(n=407)	death(n=199)	P-value
Age (years), mean (SD)	52.6±16.1	48.0±14.1	61.8±16.0	<0.001

Suppl table 2（continued）

characteristics	All patients(n=606)	Non death(n=407)	death(n=199)	P-value
Female, n (%)	222(36.6)	162(39.8)	60(30.2)	
Marriage,n(%)				
Discoverture	30(5.0)	22(5.4)	8(4.0)	0.460
Married	576(95.1)	385(94.6)	191(96.0)	0.460
General Educational Development,n(%)				
Primary and below	244(40.3)	152(37.3)	92(46.2)	0.036
Primary school or above	362(59.7)	255(62.7)	107(53.8)	0.036
Health care type,n(%)				
Self-paying	90(14.9)	79(19.4)	11(5.5)	<0.001
Medicare reimbursement	517(85.3)	329(80.8)	188(94.5)	<0.001
Occupation,n(%)				
Unemployed	529(87.3)	344(84.5)	185(93.0)	0.003
Employed	77(12.7)	63(15.5)	14(7.0)	0.003
Dialysis duration（months）, mean (SD)	47.9±33.6	55.4±33.2	32.5±29.1	<0.001
Smoking history,n(%)	130(21.5)	74(18.2)	56(28.1)	0.005
BMI, （kg/m^2）, mean (SD)	22.6±3.1	22.4±3.1	22.9±2.9	0.422
SBP（mmHg）, mean (SD)	149.2±21.6	149.9±22.8	147.8±18.9	0.351
DBP（mmHg）, mean (SD)	83.6±14.4	85.4±14.6	80.0±13.2	<0.001
Daily urine volume（ml）, mean (SD)	807.5±512.5	847.5±512.1	725.6±504.9	0.006
Used dialysate volume,（L）, mean (SD)	6.0±2.1	5.8±2.2	6.5±2.1	<0.001
Daily net PD ultrafiltration	651.9±433.5	660.1±434.9	635.2±431.3	0.497

Suppl table 2（continued）

characteristics	All patients(n=606)	Non death(n=407)	death(n=199)	P-value
volume,（ml）, mean (SD)				
Total weekly Kt/V, mean (SD)	1. 8±0. 5	1. 8±0. 5	1. 9±0. 6	0. 372
eGFR(ml/min.1.73m²) , median (IQR)	4. 8[3. 7, 6. 7]	4. 6[3. 6, 6. 2]	5. 3[3. 9, 8. 0]	<0. 001
PET , mean (SD)	0. 74±0. 15	0. 74±0. 15	0. 70±0. 15	0. 138
Primary renal disease,n(%)				
Glomerulonephritis	364(60. 1)	270(66. 3)	94(47. 2)	<0. 001
Diabetes	154(25. 4)	81(19. 9)	73(36. 7)	<0. 001
Hypertension	11(1. 8)	6(1. 5)	5(2. 5)	0. 369
Others	77(12. 7)	50(12. 3)	27(13. 6)	0. 656
Hypertension,n(%)	554(91. 4)	381(93. 6)	173(86. 9)	0. 006
Charlson Comorbidities,(%)				
cci1 Coronary artery disease	59(9. 7)	25(6. 1)	34(17. 1)	<0. 001
cci2 Congestive heart failure	136(22. 4)	58(14. 3)	78(39. 2)	<0. 001
cci3 Chronic lung disease	41(6. 8)	18(4. 4)	23(11. 6)	0. 001
cci4 Peptic ulcer disease	27(4. 5)	18(4. 4)	9(4. 5)	0. 955
cci5 Peripheral vascular disease	173(28. 5)	100(24. 6)	73(36. 7)	0. 002
cci6 Hepatic disease *(unspecified)*	83(13. 7)	58(14. 3)	25(12. 6)	0. 570
cci7 Cerebrovascular disease	84(13. 9)	51(12. 5)	33(16. 6)	0. 175
cci8 Connective tissue disorder	15(2. 5)	8(2. 0)	7(3. 5)	0. 248
cci9 Diabetes	159(26. 2)	84(20. 6)	75(37. 7)	<0. 001
cci10 Dementia	3(0. 5)	1(0. 2)	2(1. 0)	
cci11 Hemiplegia or paraplegia	20(3. 3)	16(3. 9)	4(2. 0)	0. 214
cci12 Moderate or severe renal disease	603(99. 5)	407(100. 0)	196(98. 5)	

Suppl table 2（continued）

characteristics	All patients(n=606)	Non death(n=407)	death(n=199)	P-value
cci13 Diabetes with chronic complication	127(21.0)	57(14.0)	70(35.2)	<0.001
cci14 Any malignancy in five years	36(5.9)	25(6.1)	11(5.5)	0.764
cci15 Leucocythemia	0(0)	0(0)	0(0)	
cci16 Lymphoma	1(0.2)	0(0.0)	1(0.5)	
cci17 Moderate or severe liver disease	19(3.1)	8(2.0)	11(5.5)	0.018
cci18 Metastatic solid tumor	1(0.2)	1(0.2)	0(0.0)	
cci19 AIDS	0(0)	0(0)	0(0)	
CCI score, mean (SD)	5.1±2.7	4.4±2.4	6.6±2.8	<0.001
ESA users,n(%)	535(88.3)	366(89.9)	169(84.9)	0.072
Use of VDRA,n(%)	262(43.2)	190(46.7)	72(36.2)	0.014
Use of Aspirin,n(%)	24(4.0)	11(2.7)	13(6.5)	0.023
Use of Clopidogrel,n(%)	160(26.4)	84(20.6)	76(38.2)	<0.001
Use of Statins,n(%)	204(33.7)	149(36.6)	55(27.6)	0.028
Use of Beta blockers,n(%)	340(56.1)	241(59.2)	99(49.7)	0.027
Use of CCB,n(%)	526(86.8)	363(89.2)	163(81.9)	0.013
Use of ACEI,n(%)	100(16.5)	56(13.8)	44(22.1)	0.009
Use of ARB,n(%)	174(28.7)	111(27. 3)	63(31.7)	0.262
No. of antihypertensive drugs,n(%)				
One	143(23.6)	94(23.1)	49(24.6)	0.678
Two	159(26.2)	104(25.6)	55(27.6)	0.584
Three	156(25.7)	116(28.5)	40(20.1)	0.026
Four	78(12.9)	59(14.5)	19(9.5)	0.088
Five	18(3.0)	8(2.0)	10(5.0)	0.037

Suppl table 2（continued）

characteristics	All patients(n=606)	Non death(n=407)	death(n=199)	P-value
Phosphate binders use,n(%)				
Did not take medicine	314(51.8)	217(53.3)	97(48.7)	0.290
Calcium phosphorus binding agent	276(45.5)	177(43.5)	99(49.7)	0.146
Non calcium phosphorus binder	16(2.6)	13(3.2)	3(1.5)	0.224
Biochemical parameters				
Hemoglobin（g/l）, mean (SD)	86.4±19.9	88.7±19.7	81.8±19.7	<0.001
Serum albumin（g/l）, mean (SD)	30.4±7.0	31.4±7.2	28.2±6.1	<0.001
Blood urea nitrogen（mmol/l）, mean (SD)	23.9±11.2	23.7±10.5	24.4±12.4	0.707
Serum creatinine（umol/l）, mean (SD)	899.6±353.1	949.8±341.8	797.1±354.6	<0.001
Uric acid（umol/l）, mean (SD)	461.0±139.0	469.3±132.9	446.5±152.1	0.059
Serum calcium（mmol/l）, mean (SD)	2.9±0.5	2.8±0.5	3.0±0.5	<0.001
Serum phosphorus（mmol/l）, mean (SD)	1.9±0.7	1.9±0.6	1.8±0.8	0.131
Serum intact PTH（pg/ml）,median (IQR)	204.5[106.2, 349.6]	213.0[124.5,390.4]	180.0[80.2,291.9]	<0.001
LDL-C（mmol/l）, mean (SD)	2.7±1.1	2.8±1.1	2.6±1.0	0.032
HDL-C（mmol/l）, mean (SD)	1.1±0.4	1.1±0.4	1.0±0.4	<0.001
Total cholesterol（mmol/l）, mean (SD)	4.6±1.5	4.7±1.4	4.4±1.6	0.004
Triglycerides（mmol/l）, mean (SD)	1.8±1.4	1.7±1.3	1.9±1.5	0.222
Cystatin-C（mg/l）, mean (SD)	6.0±1.9	5.9±1.8	6.2±1.9	0.012
Serum ferritin（ng/ml）, mean (SD)	390.9±331.9	428.0±371.3	442.6±375.6	0.583

Suppl table 2（continued）

characteristics	All patients(n=606)	Non death(n=407)	death(n=199)	P-value
Cardiac troponin T（ng/ml）,median (IQR)	0. 04[0. 02, 0. 1]	0. 04[0. 02, 0. 09]	0. 05[0. 02, 0. 11]	0. 153
NT-proBNP（pg/ml）,median (IQR)	9948. 0[2401. 0, 33713. 0]	8945. 0[2407. 0, 28248. 0]	13478. 0[2530. 0, 35000. 0]	0. 161
PCT（ng/ml）,median (IQR)	0. 5[0. 2, 1. 9]	0. 5[0. 2, 1. 7]	0. 6[0. 2, 2. 3]	0. 267
CRP（mg/l）,median (IQR)	14. 7[4. 1, 48. 1]	14. 6[4. 3, 46. 4]	15. 0[4. 1, 49. 1]	0. 977
Echocardiographic parameters, mean (SD)				
EF（%）	59. 4±7. 5	59. 7±7. 5	59. 0±7. 5	0. 365
Left ventricular diastolic volume index (ml/m^2)	62. 7±19. 9	99. 7±31. 6	104. 2±36. 0	0. 154
Right ventricular diameter（cm）	6. 4±0. 6	3. 4±0. 5	3. 5±0. 6	0. 006
Right Atrium diameter（cm）	3. 6±1. 0	3. 6±1. 2	3. 6±0. 5	0. 697
Pulmonary artery diameter（cm）	2. 4±0. 4	2. 4±0. 4	2. 5±0. 4	0. 657
LVPWT（cm）	1. 1±0. 3	1. 1±0. 2	1. 1±0. 2	0. 767
Left ventricular diameter（cm）	5. 0±0. 7	5. 0±0. 7	5. 1±0. 6	0. 157
IVST（cm）	1. 2±0. 2	1. 2±0. 2	1. 2±0. 2	0. 375
Left atrial dimension（cm）	3. 9±0. 6	3. 8±0. 7	4. 0±0. 6	0. 039
Sinus of aorta（cm）	3. 4±0. 4	3. 4±0. 4	3. 4±0. 4	0. 468
LVMI（g/m^2）, mean (SD)	142. 0±48. 4	141. 9±48. 6	142. 1±47. 9	0. 967
Electrocardiogram ST-T change,n(%)	293(48. 4)	203(49. 9)	90(45. 2)	0. 282
LVH on ECG, n(%)	108(17. 8)	73(17. 9)	35(17. 6)	0. 916
Auricular fibrillation on ECG,n(%)	11(1. 8)	5(1. 2)	6(3. 0)	0. 122

Suppl table 2（continued）

characteristics	All patients(n=606)	Non death(n=407)	death(n=199)	P-value
Atherosclerosis on DR,n(%)	111(18.3)	78(19.2)	33(16.6)	0.440
Cardiac enlargement on DR,n(%)	127(21.0)	96(23.6)	31(15.6)	0.023

Data are presented as percentages,median(interquartile range)or mean±SD. SD, standard deviation; BMI，body mass index; SBP，systolic blood pressure；DBP，Diastolic blood pressure；PD, peritoneal dialysis; eGFR, estimated glomerular filtration rate; PET, peritoneal equilibration test; ESRD, end-stage renal disease; AIDS, acquired immunodeficiency syndrome; cci, charlson-comorbidity index; PTH ， parathyroid hormone; NT-pro-BNP, N-terminal pro-brain natriuretic peptide; ESA,erythropoiesis-stimulating agents; VDRA,vitamin D receptor activator; CCB,calcium channel blocker; ACEI, angiotension converting enzyme inhibitors; ARB, Angiotensin Receptor Blocker; LDL-C, low-density lipoprotein cholesterol; HDL-C,high-density lipoprotein cholesterol; CRP, C-reactive protein; PCT, procalcitonin; EF,ejection fraction; LVPWT,left ventricular diastolic posterior wall thickness; LVMI, Left ventricular mass index; IVST, interventricular septum thickness; ECG,electrocardiograph; LVH, Left ventricular hypertrophy; DR,Digitalradiography. P values <0.05 were considered statistically significant.

Suppl table 3. The hyperparameters of xgboost model at each endpoint

The end point	objective	learning_rate	max_depth	min_child_weight	reg_lambda
HF	binary:logistic	0.1	1	12	3
1-year follow up HF	binary:logistic	0.1	9	9	3
5-year follow up HF	binary:logistic	0.1	3	5	2
All cause death	binary:logistic	0.1	1	15	3
1-year all cause death	binary:logistic	0.1	3	12	3
5-year all cause death	binary:logistic	0.1	3	12	3

Suppl table 4. The hyperparameters of random forest model at each endpoint

The end point	criterion	max_depth	min_impurity_decrease	n_estimators
HF	gini	5	0	80

1-year follow up HF	gini	5	0	100
5-year follow up HF	gini	3	0	10
All cause death	gini	3	0	90
1-year all cause death	gini	1	0	20
5-year all cause death	gini	3	0	50

Suppl table 5. The hyperparameters of adaboost model at each endpoint

The end point	learning_rate	n_estimators
HF	0.3	10
1-year follow up HF	0.1	50
5-year follow up HF	0.3	50
All cause death	0.1	10
1-year all cause death	0.1	5
5-year all cause death	0.1	5

Suppl figure

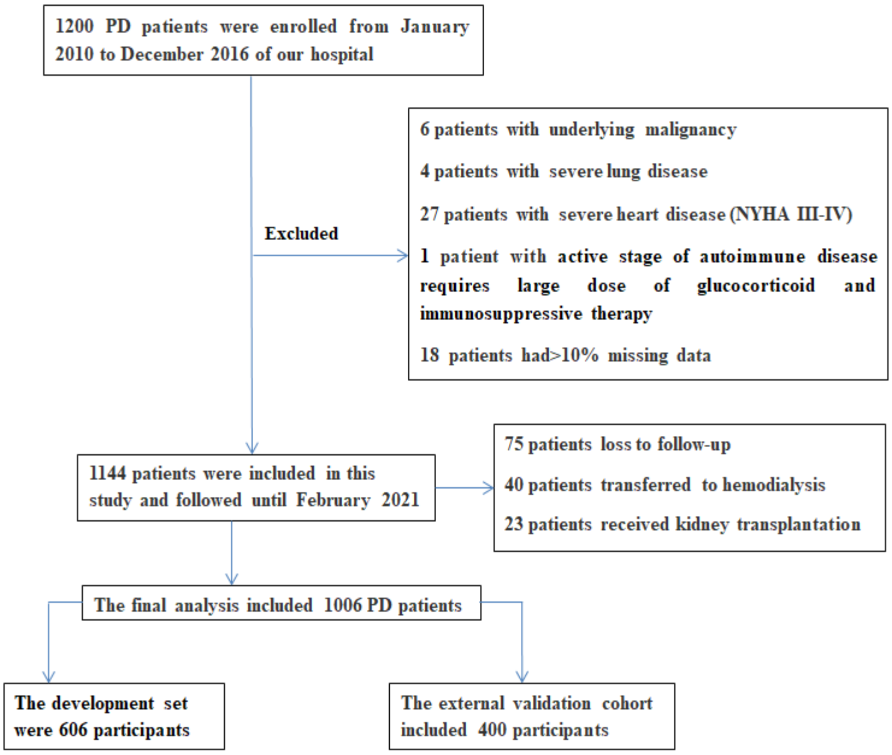

Work flow of the patients' inclusion procedure.

Suppl fig.1.Work flow of the patients' inclusion procedure.

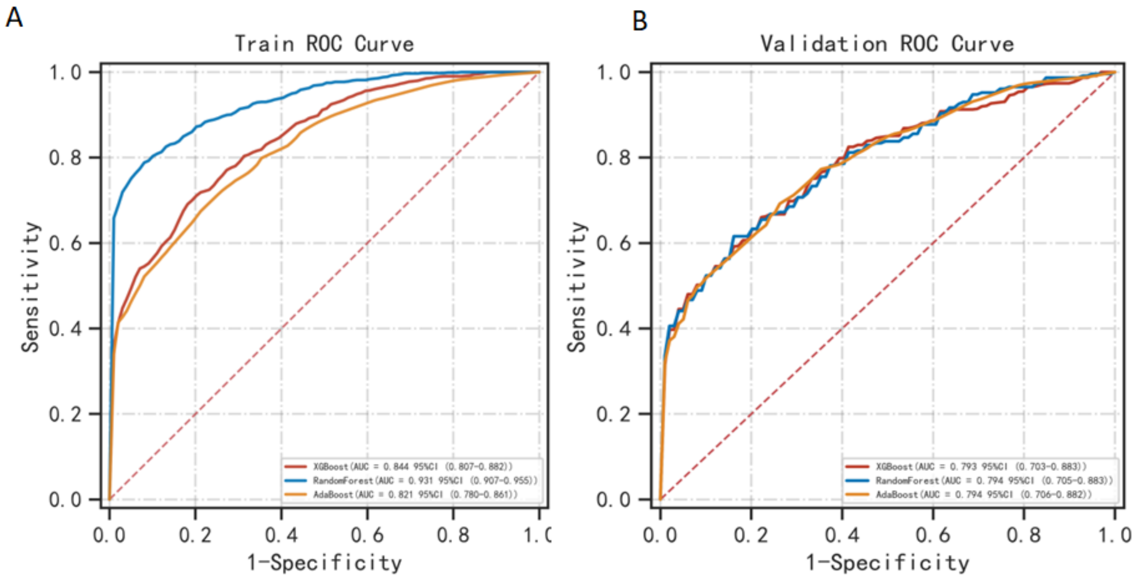

Suppl fig.2. A.The ROC curve for the xgboost、randomforest and adaboost models for predicting HF in the training set. AUC, area under the curve. B. The ROC curve for the xgboost、randomforest and adaboost models for predicting HF in the validation set. AUC, area under the curve.

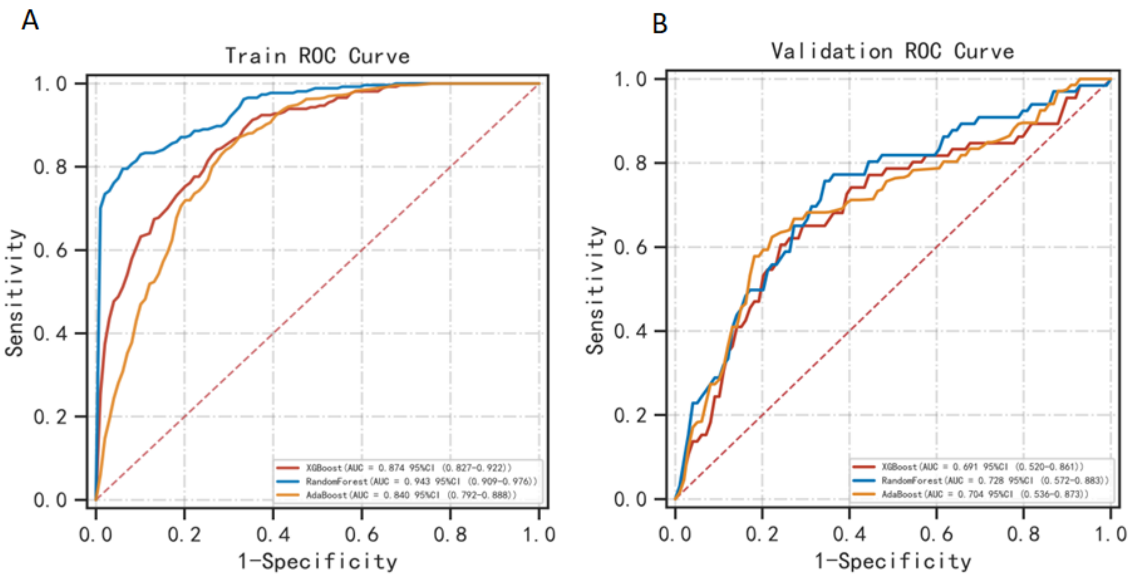

Suppl fig.3. A.The ROC curve for the xgboost、randomforest and adaboost models for predicting HF at year 1 in the training set. AUC, area under the curve. B. The ROC curve for the xgboost、randomforest and adaboost models for predicting HF at year 1 in the validation set. AUC, area under the curve.

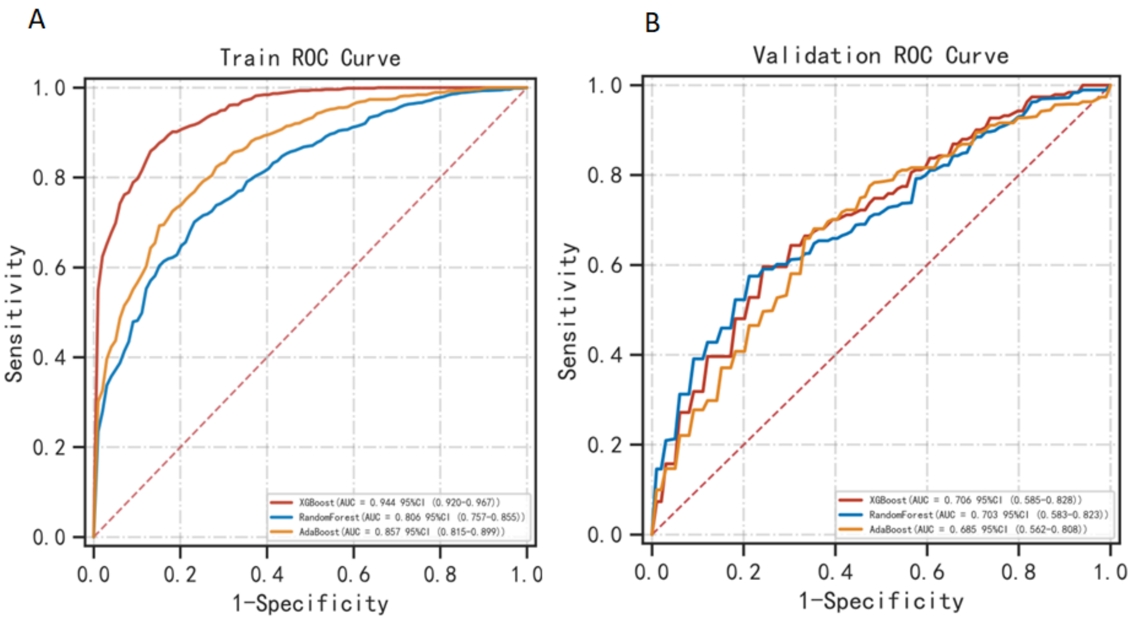

Suppl fig.4. A.The ROC curve for the xgboost、randomforest and adaboost models for predicting HF at year 5 in the training set. AUC, area under the curve. B. The ROC curve for the xgboost、random forest and adaboost models for predicting HF at year 5 in the validation set. AUC, area under the curve.

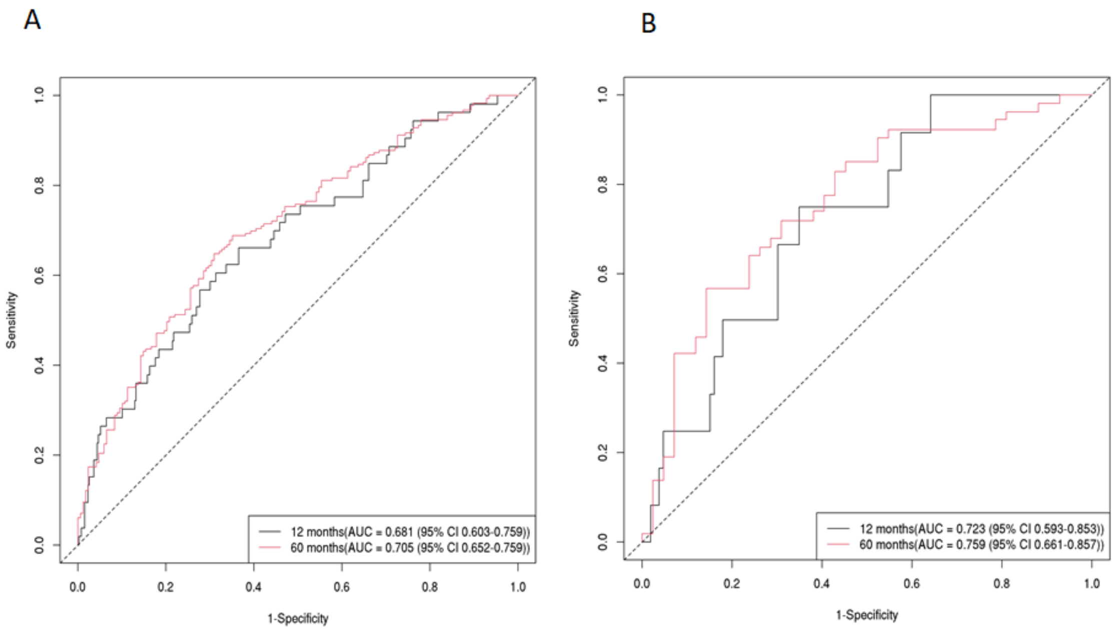

Suppl fig.5. A.The ROC curve for the cox model for predicting HF at year 1 and year 5 follow up in the training set. AUC, area under the curve. B. The ROC curve for the cox model for predicting HF at year 1 and year 5 follow up in the test set. AUC, area under the curve.

Age（yrs）	
<50	0
50-60	1.0
60-70	1.5
70-80	1.6
80-90	1.8
>90	2

BMI(kg/m2)	
<25	0
25-30	2
>30	7

SBP（mmHg）	
<100	9
100-140	0
140-160	4
>160	6

Daily urine volume（ml）	
<100	3
100-400	2
400-800	1
800-1200	0.5
>1200	0

Coronary artery disease	
Positive	9

Peripheral vascular disease	
Positive	4

Diabetes	
Positive	6

Electrocardiogram ST-T change	
Positive	4

Congestive HF	
Positive	10

Smoking history	
Positive	2

Risk Score	1-year HF Risk	Risk Score	5-year HF Risk
>38.0	>70%	>27.5	>90%
32-38	70%	20-27.5	90%
25-32	50%	14-20	70%
11-25	30%	7-14	50%
<11	<10%	<7	<30%

Suppl fig.6. The risk score system for HF.Based on a scoring system of 10 important heart failure risk variables, the risk score ranges from 0 to 56. Observed scores range from 0 to>38 points. The risk of heart failure increased with the increase of the score at 1 and 5 years of follow-up. According to the risk score, it is divided into three risk levels (<11 or <7 risk=0,

11-38 or 7-27.5 risk=1, >38 or >27.5 risk=2). BMI，body mass index; SBP, systolic blood pressure；HF, heart failure.

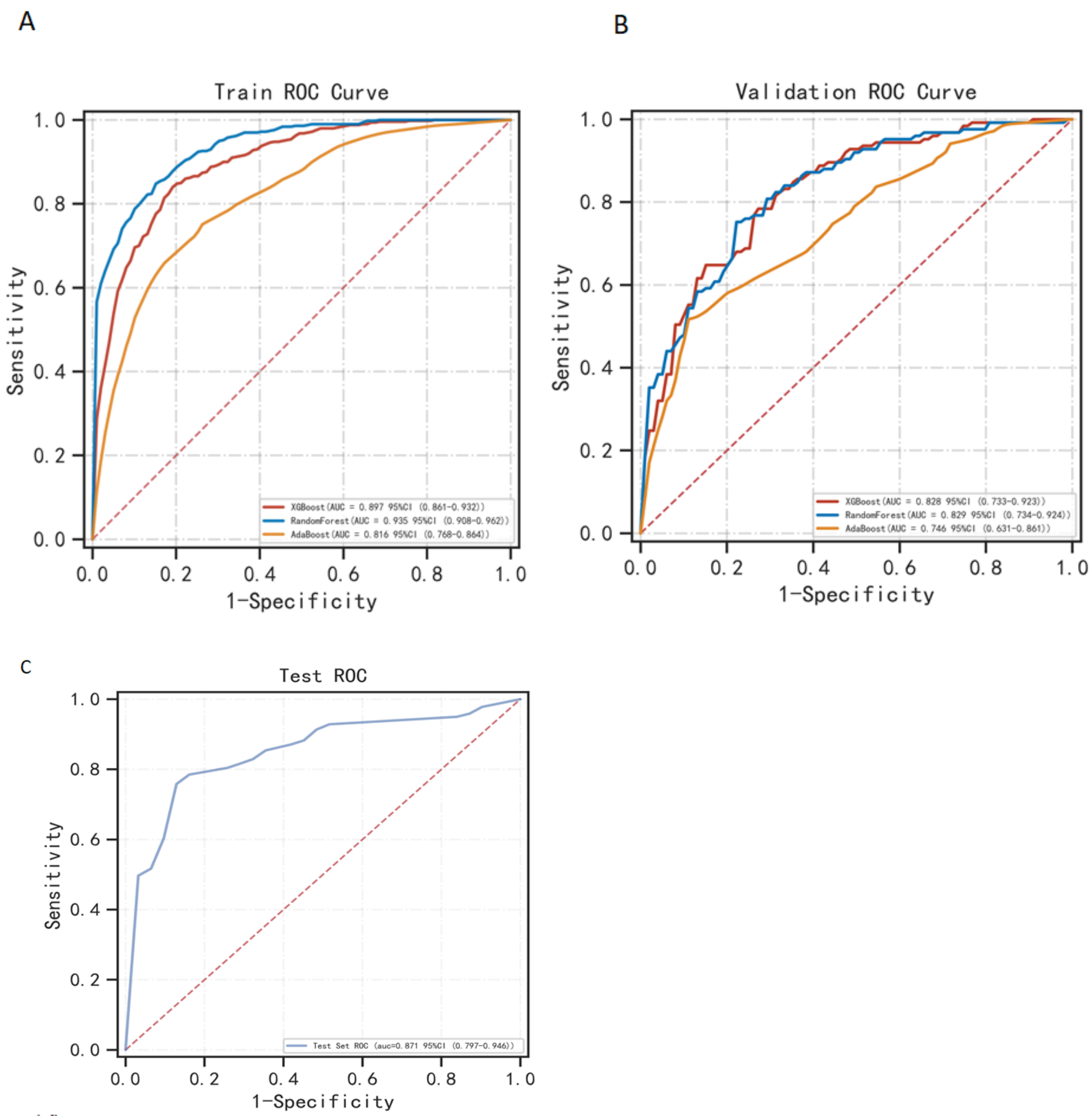

Suppl fig.7. A.The ROC curve for the xgboost、randomforest and adaboost models for predicting all-cause death in the training set. AUC, area under the curve. B. The ROC curve for the xgboost、randomforest and adaboost models for predicting all-cause death in the validation set. AUC, area under the curve. C.The ROC curve for the randomforest model for predicting all-cause death in the test set. AUC, area under the curve.

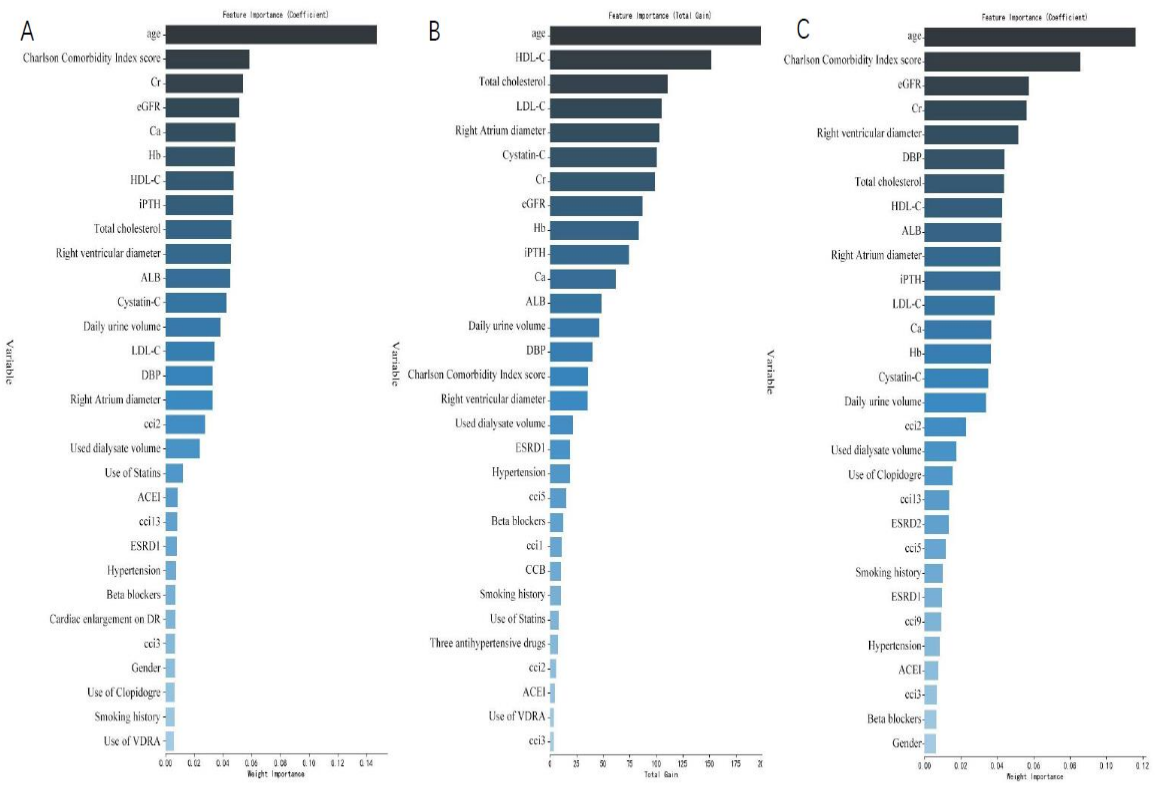

Suppl fig.8-A.B.C Variable importance analysis.Results indicate the decrease in accuracy of the final model on exclusion of each specific variable, quantified on scale are 0 to 0.14, 0 to 200, 0 to 0.12， respective. While 0 represents the minimum importance (lowest decrease in the accuracy when excluded) ,0.14, 200, 0.12 represent the maximum importance (highest decrease in the accuracy when excluded). A:Variable importance analysis of all cause death;B:Variable importance analysis of 1-year all cause death;C:Variable importance analysis of 5-year all cause death. cci1, myocardial infarction; cci2, congestive heart failure; cci3, peripheral vascular disease; cci5, dementia; cci9, mild liver disease; ESRD1, primary glomerulonephritis; ESRD2, diabetes; Cr, creatinine; eGFR, estimated glomerular filtration rate;Ca, correct calcium; Hb, hemoglobin; HDL-C, high-density lipoprotein cholesterol; iPTH, intact parathyroid hormone; ALB, serum albumin; LDL-C, low-density lipoprotein; DBP, diastolic blood pressure; ACEI, angiotension converting enzyme inhibitors; VDRA, vitamin D receptor activator.

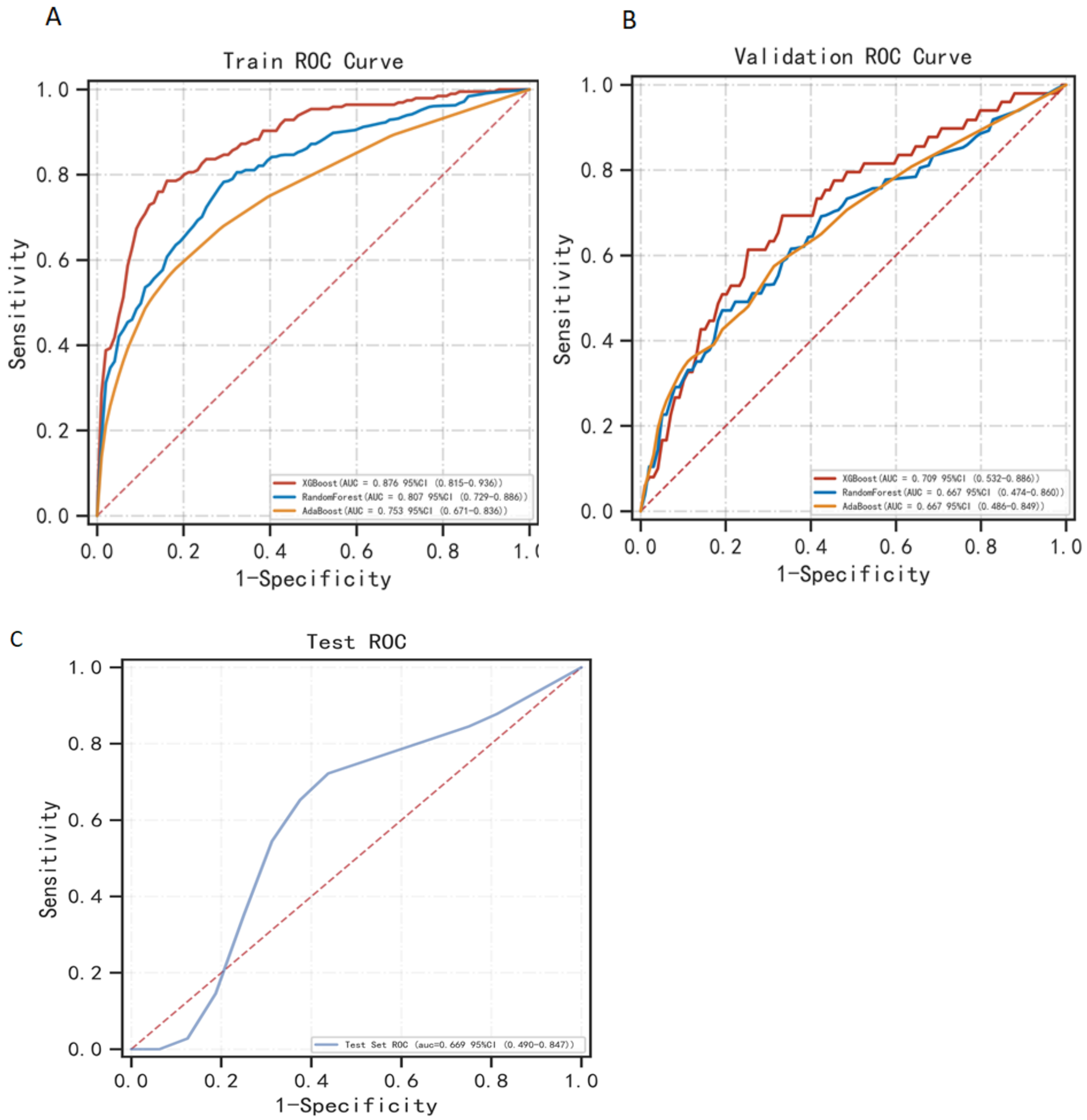

Suppl fig.9. A.The ROC curve for the xgboost、randomforest and adaboost models for predicting all-cause death at year 1 in the training set. AUC, area under the curve. B. The ROC curve for the xgboost、randomforest and adaboost models for predicting all-cause death at year 1 in the validation set. AUC, area under the curve. C.The ROC curve for the xgboost model for predicting all-cause death at year 1 in the test set. AUC, area under the curve.

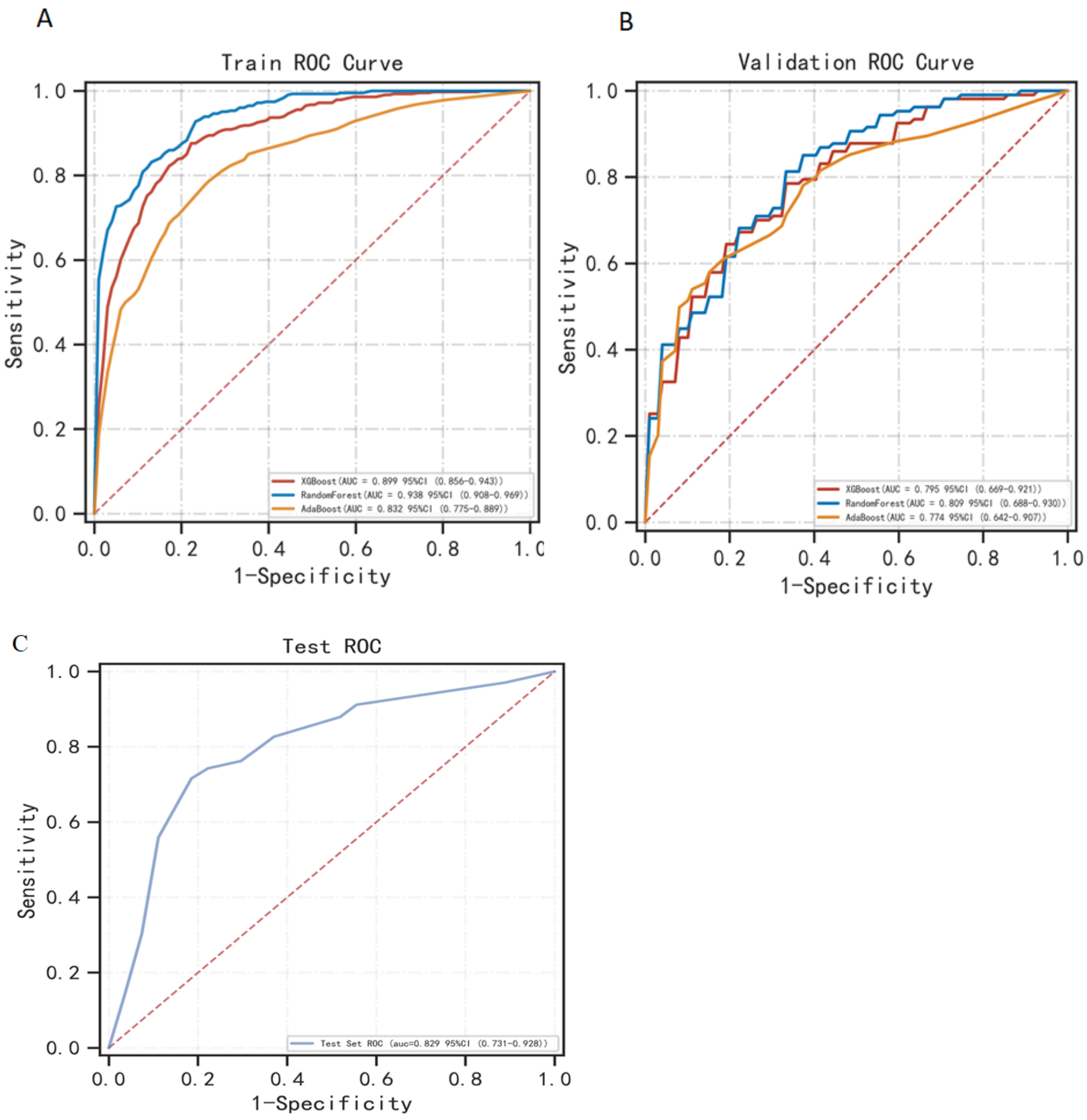

Suppl fig.10. A.The ROC curve for the xgboost、randomforest and adaboost models for predicting all-cause death at year 5 in the training set. AUC, area under the curve. B. The ROC curve of xgboost、randomforest and adaboost models for predicting all-cause death at year 5 in the validation set. AUC, area under the curve. C.The ROC curve of the randomforest model for predicting all-cause death at year 5 in the test set. AUC, area under the curve.

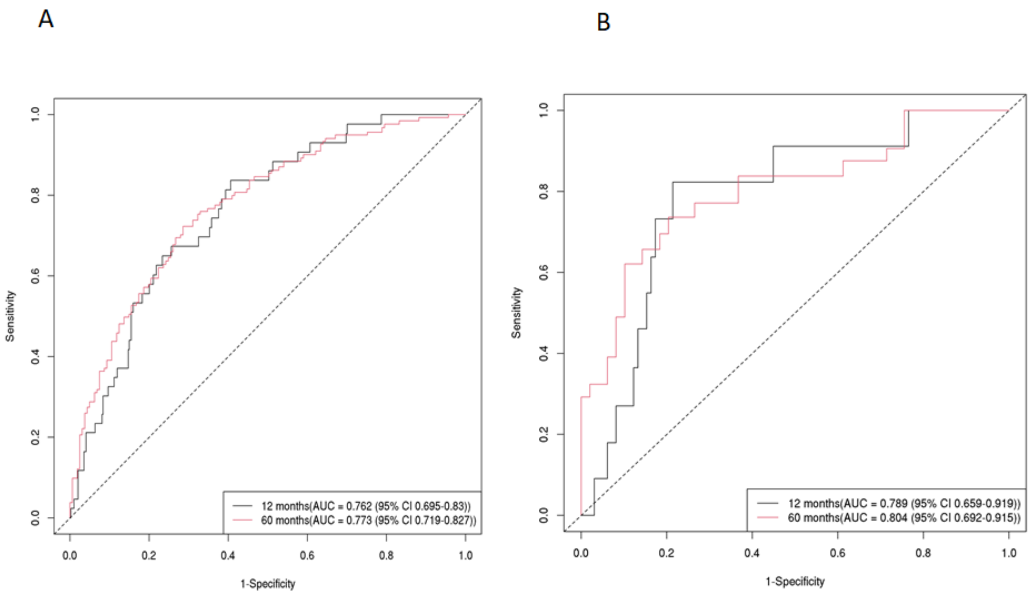

Suppl fig.11. A.The ROC curve for the cox model for predicting all cause death at year 1 and year 5 follow up in the training set. AUC, area under the curve. B. The ROC curve for the cox model for predicting all cause death at year 1 and year 5 follow up in the test set. AUC, area under the curve.
